# Mass Spectrometry-Based Metabolite Profiling in the Mouse Liver following Exposure to Ultraviolet B Radiation

**DOI:** 10.1371/journal.pone.0109479

**Published:** 2014-10-02

**Authors:** Hye Min Park, Jong Cheol Shon, Mee Youn Lee, Kwang-Hyeon Liu, Jeong Kee Kim, Sang Jun Lee, Choong Hwan Lee

**Affiliations:** 1 Department of Bioscience and Biotechnology, Konkuk University, Gwangjin-gu, Seoul, Republic of Korea; 2 College of Pharmacy and Research Institute of Pharmaceutical Sciences, Kyungpook National University, Buk-gu, Daegu, Republic of Korea; 3 Food Research Institute, AmorePacific Corporation R&D Center, Giheung-gu, Yongin, Gyeonggi-do, Republic of Korea; Korea University, Republic of Korea

## Abstract

Although many studies have been performed on the effects of ultraviolet (UV) radiation on the skin, only a limited number of reports have investigated these effects on non-skin tissue. This study aimed to describe the metabolite changes in the liver of hairless mice following chronic exposure to UVB radiation. We did not observe significant macroscopic changes or alterations in hepatic cholesterol and triglyceride levels in the liver of UVB-irradiated mice, compared with those for normal mice. In this study, we detected hepatic metabolite changes by UVB exposure and identified several amino acids, fatty acids, nucleosides, carbohydrates, phospholipids, lysophospholipids, and taurine-conjugated cholic acids as candidate biomarkers in response to UVB radiation in the mouse liver by using various mass spectrometry (MS)-based metabolite profiling including ultra-performance liquid chromatography-quadrupole time-of-flight (TOF)-MS, gas chromatography-TOF-MS and nanomate LTQ-MS. Glutamine exhibited the most dramatic change with a 5-fold increase in quantity. The results from altering several types of metabolites suggest that chronic UVB irradiation may impact significantly on major hepatic metabolism processes, despite the fact that the liver is not directly exposed to UVB radiation. MS-based metabolomic approach for determining regulatory hepatic metabolites following UV irradiation will provide a better understanding of the relationship between internal organs and UV light.

## Introduction

Ultraviolet (UV) radiation exerts a few beneficial effects (e.g., vitamin D production and use in treatment for jaundice, psoriasis, eczema, and vitiligo) but it also induces harmful responses such as sunburn, tanning, premature skin aging, suppression of the immune system, damage to the eyes, and cancer [1]–[4]. UV light is composed of UVA (320–400 nm), UVB (280–320 nm), and UVC (200–280 nm) light. Depending on its wavelength, UV light penetrates the skin and interacts with different cells located at different depths. UVB light is mostly absorbed in the epidermis of the skin and acts on DNA through direct excitation of its aromatic heterocyclic nucleobases. It causes direct damage to DNA, initiating the formation of the photoproducts cyclobutane pyrimidine dimers (CPDs) and pyrimidine pyrimidone (6-4PP). In addition to the direct effects of UVB radiation on DNA, UVB rays indirectly cause the production of free radicals, including reactive oxygen and nitrogen species [5]–[8]. The majority of previous studies on UV light have focused on the effects of acute/chronic exposure in skin tissue by using biochemical and molecular biological techniques. We also recently reported mass spectrometry (MS)-based metabolite profiling for time-dependent skin biomarkers in UVB-irradiated mice [9]. However, only a few reports have actually described the effects of UV radiation on non-skin tissues. Recently, Svobodová *et al.* [10] demonstrated that acute exposure to UVA/UVB light results in significant changes in oxidative stress-related biomarkers in the skin, liver, and blood of SKH-1 hairless mice. The liver has several functions; it regulates the levels of most chemicals in the blood, excretes bile, and detoxifies harmful compounds. Although the liver is not directly exposed to UV light, superoxide dismutase (SOD) activity and glutathione (GSH) levels are significantly altered following acute UVB exposure [10]. However, no study has yet been performed on metabolite alterations caused by chronic UVB exposure in the liver.

Identifying liver metabolites that change after exposure to UV is necessary to understand the indirect effect of UV irradiation on the liver in detail. Metabolites are critical in biology due to their involvement in cellular and physiological energetics, structure, and signaling [11]. Recently, metabolite profiling of the liver from mouse and human models was performed using nuclear magnetic resonance (NMR) and MS-based high-throughput techniques. The use of analytical instrument alone may not be sufficient because each instrument is limited in the chemical species it can analyze. Thus, the combined application of various analytical instruments is better suited to fully understand the correlation between liver and altered metabolites by analyzing the several types of compounds. Together with the use of MS, multivariate statistical analysis, such as principal component analysis (PCA), partial least-squares (PLS)-discriminant analysis (DA), and orthogonal PLS (OPLS)-DA, examines the relationship between experimental groups and involves data with several variables [12]–[14].

In the current study, the metabolites in the mouse liver after UVB irradiation for 6 weeks were profiled using various MS-based techniques including ultra-performance liquid chromatography (UPLC)-quadrupole time-of-flight (Q-TOF)-MS, gas chromatography (GC)-TOF-MS and nanomate LTQ-MS analyses with multivariate statistical analysis. We tentatively identified metabolites involved with various liver metabolism processes suggesting the hypothesis that could be used to distinguish liver tissue of non-exposed and UVB-exposed mice.

## Materials and Methods

### Reagents

Methanol, water, and acetonitrile were purchased from Fisher Scientific (Pittsburgh, PA, USA). Formic acid, dichloromethane, methoxyamine hydrochloride, and *N*-methyl-*N*-(trimethylsilyl)trifluoroacetamide (MSTFA) were obtained from Sigma Chemical Co. (St. Louis, MO, USA). All chemicals and solvents were of analytical grade and are commercially available.

### Animals

Six-week-old female albino hairless mice (Skh:hr-1) weighing 18–22 g were obtained from Charles River Laboratories (Seoul, Korea). The animals were acclimatized for 1 week in an animal facility prior to the experiments and housed under controlled conditions of temperature (23±2°C), relative humidity (55% ±10%), and 12-hr light/dark light. The animals had free access to the laboratory diet (Purina, Seoul, Korea) and ion-sterilized tap water. All animal experiments were approved by the Amorepacific Institutional Animal Care and Use Committee (AP11-009-PE007) and adhere to the OECD guidelines.

### Experimental design

The hairless mice were divided into the following two groups, with 10 mice in each group: normal group and UVB group. Each group of ten mice was housed in a cage. The average amount of feed consumed daily in each group was calculated statistically as mice were individually weighed once per week through the entire experimental period. The hairless mice of the normal group were sham irradiated, while those of the UVB group were exposed to UVB radiation 3 times per week (12 AM) starting with 1 minimal erythema dose (MED, 1 MED = 55 mJ/cm^2^) for the first week. Then, the intensity was increased by 1 MED per week for up to 4 weeks, after which the mice were exposed to 4 MED for the duration of the experiment. The mice could move around freely in the cage during the period of exposure in a steel irradiation chamber. To mimic UV rays from sun, we used 10 fluorescent lamps (TL 20W/12RS; peak emission, 320 nm; wavelength, 275–390 nm; Philips, Amsterdam, Netherlands), and the UVB emission was monitored with a UV radiometer (VLX-3W; Vilber Lourmat, France). The irradiation intensity was measured at the bottom of the cage. After exposing mice to UVB radiation during week 6, mice of each group were sacrificed by cervical dislocation and liver tissue samples were collected.

### Biochemical analyses

The hepatic cholesterol and triglyceride (TG) concentrations were determined with commercial kits (Abcam plc, Cambridge, UK). The statistical analysis was performed by an independent *t*-test.

### Sample preparation

The liver extracts were prepared according to the modified method by Masson *et al*. [15] The mixture solvent (1.2 mL) with MeOH and water (1∶1, v/v) was added to the frozen liver tissues (about 100 mg), which were then homogenized (30 frequency) for 2 min using a mixer mill (MM400; Retsch, Haan, Germany). The suspension was centrifuged at 4°C and 13,500 *g* for 10 min, and the resulting supernatant (MeOH/water (MW) extracts) was transferred to a 2 mL microcentrifuge tube. The remaining pellets were extracted again with 1 mL of the mixture solvent (dichloromethane:MeOH, 3∶1, v/v). The supernatants (dichloromethane/MeOH (DM) extracts) were collected in new microcentrifuge tubes following centrifugation. Each extract solution was evaporated with a speed-vacuum machine. Dried samples were stored at −80°C until UPLC-Q-TOF-MS and GC-TOF-MS analyses. Dried samples were resuspended with methanol/water (1∶1, v/v) and were filtered through a 0.2-µm PTFE filter for the UPLC-Q-TOF-MS analysis. For GC-TOF-MS analysis, liver tissue extracts were oximated with 50 µL of methoxyamine hydrochloride (20 mg/mL) in pyridine at 30°C for 90 min. As a second derivatizing agent, 50 µL of MSTFA was added to the mixture, which was then incubated at 37°C for 30 min. Each liver extract was prepared with the same concentration for normalization of the different amount of tissue. The final concentration of each analyzed sample was 5 mg/mL.

For nanomate LTQ-MS analysis, total lipids in liver sample were extracted using a standard Bligh and Dyer’s method. Liver (150 mg) was homogenized in the Tissue Lyser (frequency 1/s : 30, 3 min, 1 time, Qiagen) with 900 µL chloroform/methanol (1∶2, v/v) and kept at room temperature for 1 h. Phase separation was achieved by adding 300 µL chloroform and 450 µL water, and the mixture was centrifuged at 10 min at 4°C and 100 *g*. The lower organic phase was transferred into a clean tube. The upper aqueous phase was reextracted with 600 µL chloroform and the mixture was centrifuged at 10 min at 4°C and 100 *g*. The resultant lower phase was combined with the previous organic phase extracts, and dried under a gentle stream of nitrogen. Dried samples were resuspended in 100 µL of chloroform/methanol (1∶9, v/v) and diluted 10-fold with chloroform/methanol (1∶9, v/v) containing 7.5 mM ammonium acetate. Aliquots were subjected to the direct infusion nanoelectrospray tandem mass spectrometry system to profile lipids in the samples.

### GC-TOF-MS analysis

GC-TOF-MS analysis was performed on an Agilent 7890 GC system (Agilent, Atlanta, GA) coupled with a Pegasus HT TOF-MS (Leco Corp., St. Joseph, MI, USA) using an Agilent 7693 autosampler (Agilent, Atlanta, GA). The system was equipped with an Rtx-5MS column (29.8 m × 0.25 mm i.d.; particle size of 0.25 µm; Restek Corp., Bellefonte, PA, USA). The front inlet and transfer line temperatures were set at 250°C and 240°C, respectively. The helium gas flow rate through the column was 1.5 mL/min, and ions were generated by a −70 eV electron impact (EI). The ion source temperature was set at 230°C, and the mass range was 50–800 *m/z*. Column temperature was maintained isothermally at 75°C for 2 min, increased to 300°C at a rate of 15°C/min, and then held constant at 300°C for 3 min. One microliter of reactant was injected into the GC-TOF-MS with a split ratio of 10∶1.

### UPLC-Q-TOF-MS analysis

UPLC-Q-TOF-MS was performed on a Waters Q-TOF Premier system (Micromass MS Technologies, Manchester, UK) with a Waters Acquity UPLC System (Waters Corp., Milford, MA, USA) that was equipped with a Waters Acquity HPLC BEH C_18_ column (100 × 2.1 mm., i.d.; particle size of 1.7 µm). The samples were separated using a linear gradient consisting of water (A) and acetonitrile (B) with 0.1% v/v formic acid under the following conditions: 5% B for 1 min, gradually increased to 100% B for 10 min, held at 100% B for 1 min, decreased to 5% B over 1 min, and finally held at 5% B for 1 min. The injection volume of samples was 5 µL, and the flow rate was maintained at 0.3 mL/min. The TOF-MS data was collected in the range of 100–1,000 *m/z* with a scan time of 0.2 s and interscan time of 0.02 s in the negative ion mode. The capillary and cone voltages were set at 3.0 kV and 60 V, respectively. The desolvation gas flow was set to 600 L/h at a temperature of 200°C, and the cone gas flow was set to 50 L/h. The ion source temperature was 200°C.

### Direct infusion-MS analysis

Liver lipid profiling was performed on a LTQ XL mass spectrometer (Thermo Fischer Scientific, West Palm Beach, FL) equipped with an automated nanospray source (TriVersa Nanomate, Advion Biosciences, Ithaca, NY) using nanoelectrospray chips with 5.5-µm diameter spraying nozzles. The ion source was controlled using the Chipsoft 8.3.1 software (Advion Biosciences). Ionization voltage was −1.4 kV in negative mode and backpressure was set at 0.4 psi. Ion transfer capillary temperature and tube voltage were 200°C and 100 V, respectively. For the lipid analysis, five microliters of each sample were loaded into a 96-well plate (Eppendorf, Hamburg, Germany), and placed on the Nanomate cooling plate, which was set to 5°C to prevent solvent evaporation. Full scan spectra were collected at the *m/z* 400–1,000 in positive ion mode. The mass spectra of each sample were acquired in profile mode over 2 min. A collision-induced dissociation (CID) was performed with over an isolated width of 3 *m/z* units, with 35% collision energy. The tandem mass spectrometry (MS/MS) triggering threshold was set to 1,000, with a default charge state of 1. All spectra were recorded with the Thermo Xcalibur software (version 2.1., Thermo Fisher Scientific). MS/MS spectra were analyzed for the identification of lipid species using LipidBlast [16] and in-house library.

### Data processing and multivariate statistical analysis

Data processing for GC-TOF-MS and UPLC-Q-TOF-MS was performed using ChromaTOF software (Leco Corp., St. Joseph, MI, USA) and MassLynx software, respectively, and raw data files were converted to the network common data form (netCDF, *.cdf). After conversion, the MS data were processed using the Metalign software package (http://www.metalign.nl) to obtain a data matrix containing retention times, accurate masses, and normalized peak intensities. Metalign parameters were set according to the specific scaling requirements as follows: a peak slope factor of 1.0, peak threshold factor of 2, peak threshold of 500 or 1,000, and average peak width at half height of 10, which corresponds to a retention time of 3–20 min and mass range of 50–800 for GC-TOF-MS; a peak slope factor of 1.0, peak threshold factor of 2, peak threshold of 3, and average peak width at half height of 10, which corresponds to a retention time of 1–10 min and mass range of 100–1,000 for UPLC-Q-TOF-MS. The resulting data were exported to Microsoft Excel (Microsoft, Redmond, WA, USA).

For nanomate LTQ-MS data, the nominal ion mass spectra, which arranged the scans between 0.5–1.0 min, was extracted using Xcalibur software (ThermoFisher Scientific, San Jose, CA, USA). We excluded the *m/z* values that showed peak intensities below 300. To normalize the spectrum, the average of the sum of intensities from the QC samples was divided by the sum of the intensities of each sample spectra, and then each value (fold) was multiplied by the intensity of each lipid species in that sample. The resulting data were exported to Microsoft Excel (Microsoft, Redmond, WA, USA).

Multivariate statistical analysis was performed using SIMCA-P+ software (version 12.0, Umetrics, Umea, Sweden). PCA, PLS-DA and OPLS-DA were performed to obtain information on differences in the metabolite profiles between two groups. The potential variables were selected based on variable importance in the projection (VIP) value (>0.7) that estimates the importance of each variable in the projection used in a PLS or OPLS model and *p* value (<0.05) using SIMCA-P+ software and Statistica 7 (StatSoft Inc., Tulsa, OK, USA). *P*-value was determined by single sample t-test for normality of two groups and Student’s t-test for significance between two groups. Following multivariate statistical analysis, the corresponding peaks as selected variables were confirmed in the original chromatogram and were positively/tentatively identified using either commercial standard compounds in comparison with the mass spectra and retention time or on the basis of the NIST mass spectral database (National Institute of Standards and Technology, FairCom, Gaithersburg, MD, USA), in-house library, and references for GC-TOF-MS. For UPLC-Q-TOF-MS, assignment of metabolites contributing to the observed variance was performed by elemental composition analysis software with the calculated mass, mass tolerance (mDa and ppm), double bond equivalents (DBEs), and iFit algorithm implemented in the MassLynx and by commercial standard compounds, the Human Metabolome Database (HMDB, http://www.hmdb.com) and Lipid Maps Database (http://www.lipidmaps.org).

## Results

### Animal characteristics

Each female hairless mouse weighed between 27.59±1.41 and 28.16±2.05 g before the study began and was then fed ad libitum. Throughout the 6 weeks of the study, all animals appeared in good shape and gained weight without significant change. Exposure to UVB irradiation led to slight increases in the hepatic cholesterol and triglycerides compared with those for the normal mice, but there were no significant differences between the groups (**Table 1**).

**Table 1:** Metabolic parameters of hairless mice exposed to non-UVB and UVB irradiation for 6 weeks.

	Group	
	normal	UVB
**Triglyceride (mM/g)**	7.02±2.29	8.61±4.84
**Total cholesterol (mg/g)** a	3.04±0.21	3.17±0.32

a The assay detects total cholesterol, including cholesterol and cholesteryl esters.

Data were presented as mean ± SD. The statistical analysis was performed by an independent *t*-test.

### Metabolite profiling of the livers from mice exposed to UVB irradiation

**Figure 1:**
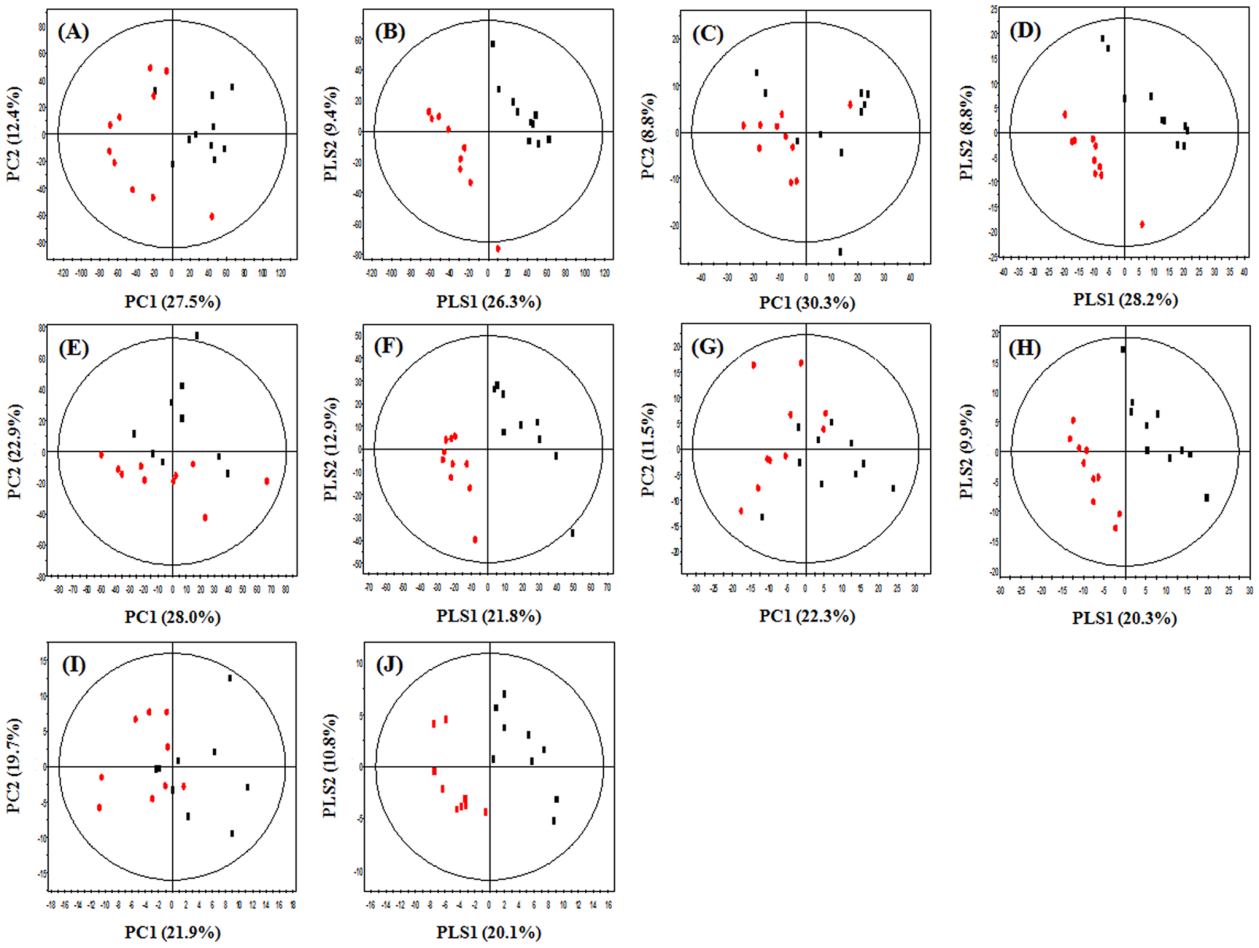
PCA score plot (A, C, E, G, I) and PLS-DA score plots (B, D, F, H, J) derived from GC-TOF-MS (A, B, E, F), UPLC-Q-TOF-MS (C, D, G, H) and Nanomate LTQ-MS (I, J) data sets for MW (A–D), DM (E–H) and lipid (I, J) extracts of mouse liver tissue after the exposure to UVB radiation for 6 weeks. ▪ - normal, • - UVB.

**Figure 2:**
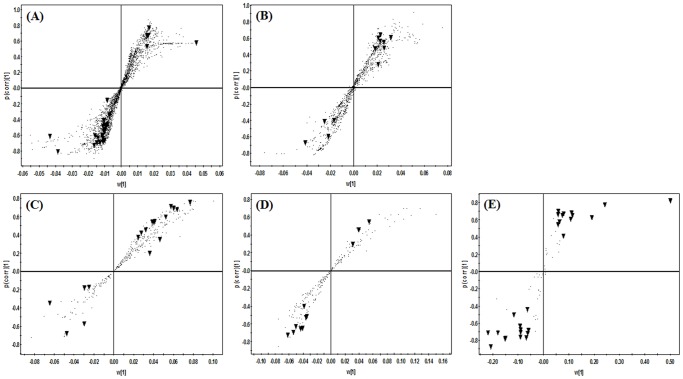
S-plots associated with OPLS-DA score plots derived from GC-TOF-MS (A, B), UPLC-Q-TOF-MS (C, D) and nanomate LTQ-MS (E) data sets for MW (A, C), DM (B, D) and lipid (E) extracts of mouse liver tissue after the exposure to UVB radiation for 6 weeks. The selected variables (▾, VIP>0.7 and *p*<0.05) are highlighted in S-plots. Each metabolites presented by a inverted triangle (▾) were the same metabolites presented in **Tables 3** **–** **5**.

In this study, we profiled the changes seen in mice hepatic metabolites on UVB exposure by using UPLC-Q-TOF-MS, GC-TOF-MS and nanomate LTQ-MS with multivariate statistical analysis. According to analytical GC and UPLC, 7,664 (MW extract) and 3,040 (DM extract) variables were detected in both the normal and UVB group in GC while 836 (MW extract) and 571 (DM extract) variables were detected in UPLC, respectively. For lipid profiling, 168 variables were detected in two experimental groups in nanomate LTQ. These variables were applied to PCA, PLS-DA score plots (**Fig. 1** and **Table S1 in File S1**) and OPLS-DA score plots to identify discriminable variables between the two experimental groups (**Fig. S1 in File S1**). Both groups were separated from each other by t [1], the predictive component, and t_0_ [1], the first orthogonal component, based on the model of R^2^X_cum_ and R^2^Y_cum_ values of 0.38–0.61 and 0.81–1.00, respectively, and with Q^2^Y_cum_ values of 0.59–0.84 in OPLS-DA model (**Table 2**). S-plots were generated using pareto scaling to visualize the metabolites responsible for the separation between groups, selected on the basis of the VIP value (>0.7) and *p* value (<0.05), respectively (**Fig. 2**).

**Table 2:** Summary of parameters for assessment of the quality of OPLS-DA models.

	Extracts	R^2^X_cum_ a	R^2^Y_cum_ a	Q^2^Y_cum_ b	*P* c
**GC-TOF-MS**	**MW** d	0.417	0.993	0.843	0.001
	**DM** e	0.379	0.984	0.834	<0.001
**UPLC-Q-TOF-MS**	**MW**	0.606	1.000	0.737	0.090
	**DM**	0.466	1.000	0.734	0.022
**Nanomate LTQ-MS**	**Lipid** f	0.521	0.812	0.587	0.015

a R^2^X_cum_ and R^2^Y_cum_ are the cumulative modeled variation in X and Y matrix, respectively.

b Q^2^Y_cum_ is the cumulative predicted variation in Y matrix.

c *P* is *p* value obtained from cross validation ANOVA of OPLS-DA.

d MW, methanol/water (1∶1, v/v).

e DM, dichloromethane/methanol (3∶1, v/v).

f The lipid extract for nanomate LTQ-MS analysis was prepared as mentioned in M&M section.

### Metabolites contributing to the discrimination between groups from the livers of mice exposed to UVB irradiation

All metabolites, including 42 metabolites in GC-TOF-MS analysis, 33 metabolites in UPLC-Q-TOF-MS analysis and 28 metabolites in nanomate LTQ-MS analysis determined by the VIP and *p* values were significantly affected by the exposure to UVB radiation for 6 weeks. In addition, the number and changes of liver metabolites by UVB exposure were generally greater in MW extracts than in DM extracts from GC and UPLC. Among them, 686 metabolites were tentatively identified as important hepatic metabolites for determining the difference between normal and UVB-irradiated mice and are summarized in **Tables 3** **–** **5**.

**Table 3:** Metabolites in MW and DM extracts from the mouse liver that were significantly different between the normal and UVB groups after 6 weeks and were tentatively identified using GC-TOF-MS analysis.

t_R_ (min)^a^	Identified ion (m/z)	Metabolites	Derivatized	Fold Changes^b^	VIP^c^	*P*-value	Related metabolism	ID^d^
5.25	116	L-Alanine	(TMS)_2_	0.68	1.48	<0.001	Amino acid metabolism	STD/MS^e^
6.71	171	Urea	(TMS)_2_	1.93	3.80	0.004	Urea cycle	STD/MS
7.06	205	Glycerol	(TMS)_3_	1.31	1.14	0.001	Glycerolipid, Carbohydrate metabolism	STD/MS
7.68	245	Fumaric acid	(TMS)_2_	0.52^DM^	2.29^DM^	0.006	TCA cycle	STD/MS
8.91	179	Nicotinamide	TMS	1.16	0.88	0.001	Nicotinate and nicotinamide metabolism	STD/MS
9.25	232	Aspartic acid	(TMS)_3_	1.36	1.30	<0.001	Amino acid metabolism	STD/MS
9.30	156	Pyroglutamic acid	(TMS)_2_	1.22	0.84	0.024	Amino acid metabolism	STD/MS
10.04	246	Glutamic acid	(TMS)_3_	1.44	1.43	<0.001	Amino acid metabolism	STD/MS
10.47	326	Taurine	(TMS)_3_	0.67	1.43	<0.001	Taurine and hypotaurine metabolism	STD/MS
11.19	156	L-Glutamine	(TMS)_3_	5.05	3.37	<0.001	Amino acid metabolism	STD/MS
12.02	204	Saccharide*		1.52^DM^	1.75^DM^	0.009	Carbohydrate metabolism	MS
12.08	205	Saccharide*		0.70	1.37	<0.001	Carbohydrate metabolism	MS
12.18	205	Glucose	MeOX, (TMS)_5_	0.49	4.01	0.008	Carbohydrate metabolism	STD/MS
12.67	204	Saccharide*		0.59	1.37	0.019	Carbohydrate metabolism	MS
12.93	313	Palmitic acid	TMS	1.21	0.97	0.001	Lipid metabolsim	STD/MS
13.00	204	Saccharide*		1.23	0.89	0.011	Carbohydrate metabolism	MS
13.95	337	Linoleic acid	TMS	1.63	1.39	0.008	Lipid metabolsim	STD/MS
13.98	339	Oleic acid	TMS	1.37	0.97	0.041	Lipid metabolsim	STD/MS
14.00	339	Elaidic acid	TMS	0.84^or^	1.19^or^	0.007	Lipid metabolsim	STD/MS
14.86	91	Arachidonic acid	TMS	1.16	0.72	0.037	Lipid metabolsim	STD/MS
15.10	338	*cis*-Oleamide	TMS	1.20^MW^/1.21^DM^	0.86^MW^/1.24^DM^	0.015^MW^/0.007^DM^	Lipid metabolsim	STD/MS
15.14	338	*trans*-Oleamide	TMS	1.25^MW^/1.28^DM^	0.88^MW^/1.41^DM^	0.030^MW^/0.009^DM^	Lipid metabolsim	MS
15.40	224	Uridine	(TMS)_4_	1.22	0.94	0.010	Nucleic acid metabolism	STD/MS
15.88	91	Docosahexaenoic acid	TMS	1.26	1.02	0.005	Lipid metabolsim	STD/MS
16.00	371	Monopalmitin	(TMS)_2_	1.39^MW^/1.21^DM^	1.24^MW^/1.25^DM^	0.004^MW^/0.008^DM^	Lipid metabolsim	STD/MS
16.05	217	Inosine	(TMS)_4_	1.23^MW^/1.42^DM^	0.97^MW^/1.57^DM^	0.003^MW^/0.046^DM^	Nucleic acid metabolism	STD/MS

Variables were determined by using the VIP value (>0.7) and *p*-value (<0.05) from the OPLS-DA model.

MW; MeOH/water extracts, DM; dichloromethane/MeOH extracts.

a t_R_ was the retention time.

b Fold change was calculated by dividing the mean of the peak intensity of each metabolite from UVB group relative to the normal group.

c VIP, variable important in the projection.

d ID, identification.

e Metabolites were identified using commercial standard compounds (STD) in comparison with the mass spectra (MS) and retention time.

*Saccharide was not successfully identified, but its mass fragments were similar to general mass fragments of saccharides.

**Table 4:** Metabolites in MW and DM extracts from the mouse liver that were significantly different between the normal and UVB groups after 6 weeks and were tentatively identified using UPLC-Q-TOF-MS analysis.

t_R_ (min)^a^	Tentative metabolites^b^	Measured MS (*m/z*)		M.W.^c^	HMDB Formula	Error (mDa)	Fold change^d^	VIP^e^	*P*-value
		Negative	Positive						
***Glycerophospholipid metabolism***									
8.09	LysoPC 22∶6*	612.3304	568.3361	567	C_30_H_50_NO_7_P	0.3	0.49	1.74	<0.001
8.12	LysoPE 20∶4	500.2770	502.2991	501	C_25_H_44_NO_7_P	−0.7	0.81^DM^	0.83^DM^	0.019
8.15	LysoPC 20∶4*	588.3293	544.3316	543	C_28_H_50_NO_7_P	−0.8	0.52^MW^/0.81^DM^	1.67^MW^/0.85^DM^	<0.001^MW^/0.016^DM^
8.21	LysoPC 22∶6*	612.3292	568.3396	567	C_30_H_50_NO_7_P	−0.9	0.6	1.2	0.012
8.28	LysoPC 20∶4*	588.3292	544.3357	543	C_28_H_50_NO_7_P	−0.9	0.65	1.12	0.013
8.49	LysoPC 20∶3*	590.3464	546.3596	545	C_28_H_52_NO_7_P	0.6	0.33^MW^/0.69^DM^	2.22^MW^/1.20^DM^	<0.001^MW^/0.003^DM^
8.54	LysoPC 16∶0*	540.3307	496.3435	495	C_24_H_50_NO_7_P	0.6	0.68^MW^/0.79^DM^	0.93^MW^/0.97^DM^	0.043^MW^/0.002^DM^
8.65	LysoPC 20∶3*	590.3467	546.3624	545	C_28_H_52_NO_7_P	0.9	0.46	1.52	0.006
8.74	LysoPE 16∶0	452.2762	454.2962	453	C_21_H_44_NO_7_P	−1.5	0.67^DM^	1.29^DM^	<0.001
8.79	LysoPC 16∶0*	540.3285	496.3407	495	C_24_H_50_NO_7_P	−1.6	0.77^DM^	1.04^DM^	0.002
8.80	LysoPC 18∶1*	566.3464	522.3554	521	C_26_H_52_NO_7_P	0.6	0.42	1.84	0.001
8.98	LysoPC 18∶1*	566.3470	522.3516	521	C_26_H_52_NO_7_P	1.2	0.61	1.17	0.014
8.99	LysoPE 18∶1	478.2933	480.3109	479	C_23_H_46_NO_7_P	−0.1	0.61^DM^	1.48^DM^	<0.001
***Bile acid metabolsim***									
4.78	Taurine conjugated cholic acid#	514.2838	538.2732	515	C_26_H_45_NO_7_S	−1.6	1.39^DM^	0.94^DM^	0.047^or^
5.29	Taurine conjugated cholic acid#	514.2843	538.2755	515	C_26_H_45_NO_7_S	0.4	1.25	0.86	0.008
6.00	Taurine conjugated deoxycholic acid§	498.2893	522.2805	499	C_26_H_45_NO_6_S	0.7	1.63^MW^/1.63^DM^	1.37^MW^/1.30^DM^	<0.001^MW^/0.012^DM^

Variables were selected by VIP value (>0.7) and *p*-value (<0.05) from OPLS-DA model.

MW; MeOH/water extracts, DM; dichloromethane/MeOH extracts.

FA; formic acid, LysoPC; lysophosphatidylcholine, LysoPE; lysophosphatidylethanolamine.

a t_R_ was retention time.

b Assignment of metabolites contributing to the observed variance was performed by elemental composition analysis software with calculated mass, mass tolerance (mDa and ppm), double bond equivalent (DBE), and the iFit algorithm was implemented in the MassLynx, and by either commercial standard compounds compared with the retention time and mass spectra or HMDB (The Human Metabolome Data Base (http://www.hmdb.ca/)).

c M.W.; molecular weight.

d Fold change was calculated by dividing the mean of the peak intensity of each metabolite from UVB group relative to normal group.

e VIP, variable important in the projection.

^*^Asterisk means the two forms of lysoPC, with the fatty acyl groups at positions 1 (*sn*-1) or 2 (*sn*-2) on the glycerol backbone.

# Cholic acid derivatives with taurine were not successfully identified, but it was predicted to be one of the following compounds: taurocholic acid, taurallocholic acid, tauro-b-muricholic acid, taurohyocholate, or tauroursocholic acid.

§ Deoxycholic acid derivatives with taurine were not successfully identified, but it was predicted to be one of compounds such as tauroursodeoxycholic acid, taurodeoxycholic acid ortaurochenodesoxycholic acid.

**Table 5:** Metabolites in lipid extracts from the mouse liver that were significantly different between the normal and UVB groups after 6 weeks and were tentatively identified using nanomate LTQ-MS analysis.

ID	*m/z* (+)	Adduct	Fold change^a^	VIP^b^	*p* value
LPC 16∶0	496.5	**[M+H]^+^**	0.75	2.83	0.004
LPC 18∶1	522.5	**[M+H]^+^**	0.63	1.95	0.001
LPC 18∶0	524.5	**[M+H]^+^**	0.63	2.68	<0.001
LPC 20∶4	544.4	**[M+H]^+^**	0.74	1.17	0.019
LPC 20∶3	546.5	**[M+H]^+^**	0.66	0.81	0.014
LPC22∶6	568.5	**[M+H]^+^**	0.69	1.16	0.006
PE 34∶2	716.5	**[M+H]^+^**	1.16	0.76	0.030
PC 32∶0	734.6	**[M+H]^+^**	1.10	1.02	0.028
PE 36∶4	740.5	**[M+H]^+^**	1.06	0.82	0.022
PE 36∶2	744.5	**[M+H]^+^**	1.11	0.75	0.043
PC 32∶0	756.6	**[M+Na]^+^**	1.21	1.44	0.005
PC 34∶2	758.6	**[M+H]^+^**	1.23	6.49	<0.001
PE 38∶6	764.5	**[M+H]^+^**	1.18	3.14	<0.001
PE 38∶5	766.5	**[M+H]^+^**	1.09	1.49	0.006
PC 34∶6	772.6	**[M+Na]^+^**	1.13	1.04	0.002
PC 34∶3	778.6	**[M+Na]^+^**	1.15	0.75	0.005
PE 39∶6		**[M+H]+**			
PE 38∶5	788.7	**[M+Na]^+^**	0.93	1.19	0.030
PE 39∶0	790.5	**[M+H]^+^**	1.11	1.40	0.005
PC 35∶1	796.6	**[M+Na]^+^**	1.20	2.48	0.018
PE 39∶5	802.6	**[M+Na]^+^**	1.15	0.97	0.002
PE 39∶0	812.7	**[M+Na]^+^**	0.90	1.95	0.004
PE 40∶6	814.6	**[M+Na]^+^**	0.92	0.87	0.008
PC 38∶4	832.8	**[M+Na]^+^**	0.92	1.50	0.045
PC 38∶3	834.8	**[M+Na]^+^**	0.89	2.32	0.010
PC 38∶2	836.8	**[M+Na]^+^**	0.90	1.18	0.004
TG 56∶4	928.6	**[M+NH_4_]^+^**	0.74	0.82	0.048

Variables were selected by VIP value (>0.7) and *p* value (<0.05) from OPLS-DA models.

a Fold change was calculated by dividing the mean of the peak intensity of each metabolite from the UVB-radiated group by that of the normal group.

b VIP, variable important in the projection.

In GC-TOF-MS analysis, the levels of 19 metabolites (aspartic acid, pyroglutamic acid, glutamic acid, _L_-glutamine, urea, nicotinamide, palmitic acid, linoleic acid, oleic acid, arachinodic acid, docosahexaenoic acid, *cis*- and *trans* oleamide, monopalmitine, uridine, inosine, glycerol and 2 saccharides) significantly increased after exposure to UVB rays, whereas the levels of 7 metabolites, that is, _L_-alanine, fumaric acid, taurine, elaidic acid, glucose, and 2 saccharides decreased (**Table 3**). Of these, _L_-glutamine exhibited a 5.05-fold increase and glucose exhibited a 0.49-fold decrease. A VIP value greater than 3.0 was used to designate the major liver metabolites that contributed to discrimination between normal and UVB-irradiated mice.

UPLC-Q-TOF-MS analysis showed that the levels of 10 lysophosphatidylcholines (lysoPCs) (with two forms of C16∶0, 18∶1, 20∶3, 20∶4 and 22∶6) and of 3 lysophosphatidylethanolamines (lysoPEs) with C 16∶0, C18∶1, and 20∶4 related with glycerophospholipid metabolism significantly decreased, while 3 metabolites associated with taurine-conjugated bile acid metabolism were positively affected by UVB irradiation in the mouse liver (**Table 4**).

In direct infusion-MS analysis, the levels of 6 lysoPCs with C16∶0, C18∶0, C18∶1, C20∶3, C20∶4 and C 22∶6 significantly decreased and this result was in agreement with the result of UPLC analysis. And, the levels of 5 phosphatidylcholines (PCs) and 7 phosphatidylethanolamines (PEs) significantly increased, whereas the levels of 3 PCs and 3 PEs significantly decreased after exposure to UVB light (**Table 5**).

## Discussion

According to comprehensive MS-based metabolite profiling, we determined that several hepatic metabolites contributed to the differences seen on chronic exposure to UVB light and were associated with various metabolisms, including amino acid, lipid, glycerolipid, and nucleic acid metabolism. It is widely accepted that some of these metabolites regulate liver function and homeostasis.

The liver is a major organ for amino acid metabolism and is largely responsible for maintaining circulating amino acid homeostasis. Amino acids, including aspartate, glutamate, glutamine, glycine, and alanine, are detected at high concentrations in the liver [17]. Our data showed significant increases of most amino acid levels, especially _L_-glutamine by UVB exposure. Glutamine and glutamate metabolism are closely related with maintenance and promotion of cell function in diverse tissues and cells, especially in the liver. _L_-Glutamine is both the most abundant extracellular amino acid and the most significant nitrogen transporter between tissues, whereas glutamate is the most abundant intracellular amino acid and plays a specific role in the transamination of most amino acids, glucose homeostasis, lipid metabolism, and the regulation of the TCA cycle and urea cycle [18]–[21]. Glutamine synthetase uses glutamate and NH_3_ to synthesize glutamine in the perivenous cells of the liver. In periportal cells, _L_-glutamine, as a precursor of glutamate, is associated with urea and glucose syntheses. Furthermore, glutamine acts as a key precursor for nucleic acid and nucleotide synthesis [19]. And high glutamine use in liver induces the production of glucose and urea [22]. Considering the multiple roles of glutamine, the increase of glutamine levels in the liver from exposure to UVB may have positive or negative influences on glutamine-related liver metabolisms.

This study showed that the levels of aspartate and urea increased, whereas the fumarate levels decreased following UVB irradiation for 6 weeks. These metabolites are associated with TCA and urea cycles, which are closely linked to each other. Urea is the major end product of nitrogen metabolism in humans and mammals. For urea formation, one of the two nitrogen atoms in urea comes from oxidative deamination of glutamate while the other nitrogen atom originates from aspartate. Aspartate is regenerated from fumarate produced by the urea cycle. Fumarate is oxidized to oxaloacetate by TCA cycle enzymes and is then converted by transamination into aspartate [23]. We predict that the increase in the urea level was probably induced, in part, by the alterations in aspartate, glutamate, and fumarate. Together with these metabolites, the levels of glycerol, glucose and some of the unidentified saccharides were altered in the liver of UVB-irradiated hairless mice. The liver also plays a unique role in controlling carbohydrate metabolism by regulating glucose production as well as glucose consumption. Glucose is produced either by breaking down glycogen (glycogenolysis) or by *de novo* synthesis of glucose (gluconeogenesis) from non-carbohydrate precursors, including lactate, amino acids, and glycerol, and is underutilized as the fuel of muscles and other organs such as the brain and kidney. The concentration of glucose in the liver is related to blood glucose levels. Excess glucose in blood converts into glycogen and is then stored in the liver. When blood sugar levels drop, the liver reconverts the glycogen back to glucose [24], [25]. Although glucose levels were not measured in blood, our finding that glucose levels showed the greatest decline in the liver may affect glucose homeostasis.

In addition, the levels of glucose affect fatty acid (FA) and glycerolipid homeostasis [25], [26]. In the liver of UVB-irradiated hairless mice, several FAs, including palmitic, linoleic, oleic, arachidonic and docosahexaenoic acid, increased, except for elaidic acid, the *trans* isomer of oleic acid. Lysophospholipids (e.g., lysoPC and lysoPE) decreased in liver (**Tables 4**). And, some PCs and PEs increased whereas others decreased in liver (**Table 5**). However, the levels of total TGs, which are formed by combining glycerol with 3 FAs, and the concentration of total cholesterol, including cholesterol and cholesteryl esters, were not altered by UVB exposure. FAs are the main component of phospholipids, TGs, and cholesterol esters. FAs and TGs are primarily an energy source for most organisms. FA metabolism in the liver involves 3 main pathways, that is, catabolism by β-oxidation, synthesis from acetyl CoA, and esterification into TGs [26], [27]. Some FAs are used in the synthesis of phospholipids and eicosanoids, including prostaglandins, thromboxanes, and leukotrienes [28]. Eicosanoids, which are key mediators and regulators of inflammation, are usually generated from arachidonic acid, one of the *n*-6 polyunsaturated FAs (PUFAs). Eicosapentaenoic acid and docosahexaenoic acid as the *n*-3 PUFAs possess anti-inflammatory activity [29], [30]. Inflammation also correlates with reactive oxygen species (ROS) production. The exposure of hepatocytes to fatty acids containing palmitic and oleic acid induced increased oxidative stress through ROS generation [31]. Furthermore, SFAs, such as palmitic and stearic acid, increase the saturation of membrane phospholipids and deregulate TCA cycle metabolism, leading to ROS accumulation [32]. Recent reports demonstrated acute exposure to UVB radiation caused significant increases in oxidative stress-related parameters, SOD activity and the level of GSH in the liver of hairless mice [10]. Together with FAs, the levels of either lysophospholipids (lysoPCs and lysoPEs) or partial phospholipids (PCs and PEs) diminished following UVB exposure in the liver. PC has been shown in numerous studies to protect liver cells from damage from a variety of toxins, such as ethanol, carbon tetrachloride. PC can also be generated *via* choline pathway or methylation of PE by the enzyme PE *N*-methyltransferase. Li *et al.* [33] demonstrated that the alteration of PC/PE ratio influences membrane integrity and can lead to liver failure since the ratio of PC to PE is a key regulator of cell membrane integrity. In this study, the mouse liver of UVB group had an increased ratio of PC to PE compared to control livers. And, in particular, the changes of unsaturated forms of lysoPC and lysoPE were remarkable. Lysophospholipids are found in small amounts in most tissues. Notably, lysoPC, as a potent chemotactant, controls initiation of the adaptive cellular immune response [34] and induces pro-inflammatory cytokine production [35]. Furthermore, saturated acyl lysoPCs such as C16∶0 and C18∶0 induce inflammation while polyunsaturated acyl lysoPCs including C20∶4 and C 22∶6 inhibit lysoPC-induced inflammation *in vivo* [36]. Based on the literature for FAs, our results with the increased level of FAs and the decreased level of lysophospholipids indicate chronic exposure to UVB radiation may also be closely related to ROS production and inflammation in the liver of UVB-irradiated mice.

Uridine and inosine were altered by chronic 6-week exposure to UVB radiation. The pyrimidine precursor uridine undergoes degradation by being essentially cleared in a single pass through the liver and the formation by *de novo* synthesis in the liver. Le *et al*. [37] demonstrated that uridine modulated liver protein acetylation profiles in reference to the regulation of cellular energy metabolism in liver tissue. Inosine, a naturally occurring purine formed from the breakdown of adenosine, exerts potent anti-inflammatory effects by inhibiting the production of pro-inflammatory cytokines, both *in vitro* and *in vivo* [38], [39]. The alterations in purine and pyrimidine nucleosides in the liver of UVB-irradiated mice can directly or indirectly influence the metabolism and immune-mediated processes of the liver.

This study demonstrated that taurine levels decreased following UVB irradiation, while those of taurine-conjugated bile acids increased, suggesting the changes in taurine and its conjugated metabolites are closely associated with major hepatic metabolism pathways. Taurine, a sulfur-containing amino acid, is found in high concentrations in all mammalian tissues and is involved in various physiological processes such as osmoregulation, antioxidation, detoxification, and bile acid conjugation in the liver [40]–[43]. Primary bile acids (e.g., cholic and chenodeoxycholic acid) and secondary bile acids (e.g., deoxycholic and lithocholic acid), which are synthesized from cholesterol and primary bile acids in the liver, primarily exist as *N*-acyl conjugated forms with glycine and taurine. The proportion of bile acid conjugated with taurine correlates with hepatic taurine concentrations [44]–[46]. These bile acids normally regulate cholesterol homeostasis, glucose metabolism, lipid solubilization, and metabolic signaling [47]–[48].

By measuring metabolite changes with a combination of MS analytic techniques in the mouse liver after UVB irradiation, we revealed that chronic exposure to UVB light may affect hepatic functions in liver metabolism processes. Although the liver is not directly exposed to UVB radiation, glutamine and glutamate metabolism, glucose and lipid homeostasis, and bile acid metabolism, were indirectly impacted through altering various kinds of metabolites._ L_-Glutamine exhibited the largest changes, indicating its potential as an indirect photodamage-related biomarker in the liver. In addition, because there are only a few in-depth reports on effects of UV light in the liver, further biochemical and molecular studies on the relationship between UVB radiation and liver tissue and between UVB radiation and altered metabolites are warranted. Nevertheless, this study suggests that the MS-based metabolomic approach for determining regulatory hepatic metabolites following the exposure to UV radiation will lead to a better understanding of the correlation between liver and UV exposure.

## Supporting Information

File S1 **Supporting information. Figure S1.** OPLS-DA score plot derived from GC-TOF-MS (A, B), UPLC-Q-TOF-MS (C, D) and nanomate LTQ-MS (E) data sets for MW (A, C), DM (B, D) and lipid (E) extracts of mouse liver tissue after the exposure to UVB radiation for 6 weeks. ▪ - normal, • – UVB. **Table S1**. Summary of parameters for assessment of the quality of PLS-DA models
(DOC)

## References

[1] Piltingsrud HV, Odland LT, Fong CW (1976) An evaluation of fluorescent light sources for use in phototherapy of neonatal jaundice. Am Ind Hyg Assoc J 37: 437–444. 961603 10.1080/0002889768507489

[2] Bishop SC (1979) DNA repair synthesis in human skin exposed to ultraviolet radiation used in PUVA (psoralen and UV-A) therapy for psoriasis. Br J Dermatol 101: 399–405. 508605 10.1111/j.1365-2133.1979.tb00017.x

[3] Midelfart K, Stenvold SE, Volden G (1985) Combined UVB and UVA phototherapy of atopic eczema. Dermatologica 171: 95–98. 4043476 10.1159/000249399

[4] El-Zawahry BM, Bassiouny DA, Sobhi RM, Abdel-Aziz E, Zaki NS, et al (2012) A comparative study on efficacy of UVA1 vs. narrow-band UVB phototherapy in the treatment of vitiligo. Photodermatol Photoimmunol Photomed 28: 84–90. 22409711 10.1111/j.1600-0781.2011.00643.x

[5] Ravanat JL, Douki T, Cadet J (2001) Direct and indirect effects of UV radiation on DNA and its components. J Photochem Photobiol B 63: 88–102. 11684456 10.1016/s1011-1344(01)00206-8

[6] Trautinger F (2001) Mechanisms of photodamage of the skin and its functional consequences for skin ageing. Clin Exp Dermatol 26: 573–577. 11696060 10.1046/j.1365-2230.2001.00893.x

[7] Svobodova A, Walterova D, Vostalova J (2006) Ultraviolet light induced alteration to the skin. Biomed Pap Med Fac Univ Palacky Olomouc Czech Repub 150: 25–38. 16936899 10.5507/bp.2006.003

[8] Verschooten L, Claerhout S, Van Laethem A, Agostinis P, Garmyn M (2006) New strategies of photopro tection. Photochem Photobiol 82: 1016–1023. 16709145 10.1562/2006-04-27-ir-884.1

[9] Park HM, Shin JH, Kim JK, Lee SJ, Hwang GS, et al.. (2013) MS-based metabolite profiling reveals time-dependent skin biomarkers in UVB-irradiated mice. Metabolomics. In press Doi: 10.1007/s11306-013-0594-x.

[10] Svobodová AR, Galandáková A, Sianská J, Doležal D, Ulrichová J, et al (2011) Acute exposure to solar simulated ultraviolet radiation affects oxidative stress-related biomarkers in skin, liver and blood of hairless mice. Biol Pharm Bull 34: 471–479. 21467631 10.1248/bpb.34.471

[11] Vinayavekhin N, Homan EA, Saghatelian A (2010) Exploring disease through metabolomics. ACS Chem Biol 5: 91–103. 20020774 10.1021/cb900271r

[12] Garrod S, Humpher E, Connor SC, Connelly JC, Spraul M, et al (2001) High-resolution ^(1)^H NMR and magic angle spinning NMR spectroscopic investigation of the biochemical effects of 2-bromoethanamine in intact renal and hepatic tissue. Magn Reson Med 45: 781–790. 11323804 10.1002/mrm.1106

[13] Kim HJ, Kim JH, Noh S, Hur HJ, Sung MJ, et al (2011) Metabolomic analysis of livers and serum from high-fat diet induced obese mice. J Proteome Res 10: 722–731. 21047143 10.1021/pr100892r

[14] Kim J, Choi JN, Choi JH, Cha YS, Muthaiya MJ, et al (2013) Effect of fermented soybean product (Cheonggukjang) intake on metabolic parameters in mice fed a high-fat diet. Mol Nutr Food Res 57: 1886–1891. 23609950 10.1002/mnfr.201200700

[15] Masson P, Alves AC, Ebbels TM, Nicholson JK, Want EJ (2010) Optimization and evaluation of metabolite extraction protocols for untargeted metabolic profiling of liver samples by UPLC-MS. Anal Chem 82: 7779–7786. 20715759 10.1021/ac101722e

[16] Kind T, Liu KH, Lee do Y, DeFelice B, Meissen JK, et al (2013) LipidBlast in silico tandem mass spectrometry database for lipid identification. Nat Methods 10: 755–758. 23817071 10.1038/nmeth.2551 PMC3731409

[17] Brosnan ME, Brosnan JT (2007) The Textbook of Hepatology: From Basic Science to Clinical Practice, 3rd Edition. Wiley-Blackwell, UK. 142–149.

[18] Watford M (2000) Glutamine and glutamate metabolism across the liver sinusoid. J Nutr 130: 983S–987S. 10736366 10.1093/jn/130.4.983S

[19] Yang D, Brunengraber H (2000) Glutamate, a window on liver intermediary metabolism. J Nutr 130: 991S–994S. 10736368 10.1093/jn/130.4.991S

[20] Newsholme P, Procopio J, Lima MM, Pithon-Curi TC, Curi R (2003) Glutamine and glutamate–their central role in cell metabolism and function. Cell Biochem Funct 21: 1–9. 12579515 10.1002/cbf.1003

[21] Brosnan ME, Brosnan JT (2009) Hepatic glutamate metabolism: a tale of 2 hepatocytes. Am J Clin Nutr 90: 857S–861S. 19625684 10.3945/ajcn.2009.27462Z

[22] Watford M, Chellaraj V, Ismat A, Brown P, Raman P (2002) Hepatic glutamine metabolism. Nutrition 18: 301–303. 11934540 10.1016/s0899-9007(02)00739-6

[23] Shambaugh GE 3rd (1977) Urea biosynthesis I. The urea cycle and relationships to the citric acid cycle. Am J Clin Nutr 30: 2083–2087. 337792 10.1093/ajcn/30.12.2083

[24] Sherwin RS (1980) Role of the liver in glucose homeostasis. Diabetes Care 3: 261–265. 6993137 10.2337/diacare.3.2.261

[25] Postic C, Dentin R, Girard J (2004) Role of the liver in the control of carbohydrate and lipid homeostasis. Diabetes Metab 30: 398–408. 15671906 10.1016/s1262-3636(07)70133-7

[26] Nguyen P, Leray V, Diez M, Serisier S, Le Bloc'h J, et al (2008) Liver lipid metabolism. J Anim Physiol Anim Nutr (Berl) 92: 272–283. 18477307 10.1111/j.1439-0396.2007.00752.x

[27] Bechmann LP, Hannivoort RA, Gerken G, Hotamisligil GS, Trauner M, et al (2012) The interaction of hepatic lipid and glucose metabolism in liver diseases. J Hepatol 56: 952–964. 22173168 10.1016/j.jhep.2011.08.025

[28] Bradbury MW (2006) Lipid metabolism and liver inflammation. I. Hepatic fatty acid uptake: possible role in steatosis. Am J Physiol Gastrointest Liver Physiol 290: G194–198. 16407588 10.1152/ajpgi.00413.2005

[29] Calder PC (2006) n-3 polyunsaturated fatty acids, inflammation, and inflammatory diseases. Am J Clin Nutr 83: 1505S–1519S. 16841861 10.1093/ajcn/83.6.1505S

[30] Calder PC (2009) Polyunsaturated fatty acids and inflammatory processes: New twists in an old tale. Biochimie 91: 791–795. 19455748 10.1016/j.biochi.2009.01.008

[31] Chavez-Tapia NC, Rosso N, Tiribelli C (2012) Effect of intracellular lipid accumulation in a new model of non-alcoholic fatty liver disease. BMC Gastroenterol 12: 20. 22380754 10.1186/1471-230X-12-20 PMC3313845

[32] Leamy AK, Egnatchik RA, Young JD (2013) Molecular mechanisms and the role of saturated fatty acids in the progression of non-alcoholic fatty liver disease. Prog Lipid Res 52: 165–174. 23178552 10.1016/j.plipres.2012.10.004 PMC3868987

[33] Li Z, Agellon LB, Allen TM, Umeda M, Jewell L, et al (2006) The ratio of phosphatidylcholine to phosphatidylethanolamine influences membrane integrity and steatohepatitis. Cell Metab 3: 321–331. 16679290 10.1016/j.cmet.2006.03.007

[34] Perrin-Cocon L, Agaugué S, Coutant F, Saint-Mézard P, Guironnet-Paquet A, et al (2006) Lysophosphatidylcholine is a natural adjuvant that initiates cellular immune responses. Vaccine 24: 1254–1263. 16229929 10.1016/j.vaccine.2005.09.036

[35] Olofsson KE, Andersson L, Nilsson J, Björkbacka H (2008) Nanomolar concentrations of lysophosphatidylcholine recruit monocytes and induce pro-inflammatory cytokine production in macrophages. Biochem Biophys Res Commun 370: 348–352. 18371300 10.1016/j.bbrc.2008.03.087

[36] Hung ND, Sok DE, Kim MR (2012) Prevention of 1-palmitoyl lysophosphatidylcholine-induced inflammation by polyunsaturated acyl lysophosphatidylcholine. Inflamm Res 61: 473–483. 22252240 10.1007/s00011-012-0434-x

[37] Le TT, Ziemba A, Urasaki Y, Hayes E, Brotman S, et al (2013) Disruption of uridine homeostasis links liver pyrimidine metabolism to lipid accumulation. J Lipid Res 54: 1044–1057. 23355744 10.1194/jlr.M034249 PMC3605981

[38] Haskó G, Kuhel DG, Németh ZH, Mabley JG, Stachlewitz RF, et al (2000) Inosine inhibits inflammatory cytokine production by a posttranscriptional mechanism and protects against endotoxin-induced shock. J Immunol 164: 1013–1019. 10623851 10.4049/jimmunol.164.2.1013

[39] Liaudet L, Mabley JG, Soriano FG, Pacher P, Marton A, et al (2001) Inosine reduces systemic inflammation and improves survival in septic shock induced by cecal ligation and puncture. Am J Respir Crit Care Med 164: 1213–1220. 11673212 10.1164/ajrccm.164.7.2101013

[40] Vessey DA (1978) The biochemical basis for the conjugation of bile acids with either glycine or taurine. Biochem J 174: 621–626. 708413 10.1042/bj1740621 PMC1185955

[41] Häussinger D (2004) Neural control of hepatic osmolytes and parenchymal cell hydration. Anat. Rec. A Discov. Mol Cell Evol Biol 280: 893–900. 10.1002/ar.a.20094 15382012

[42] Refik Mas M, Comert B, Oncu K, Vural SA, Akay C, et al (2004) The effect of taurine treatment on oxidative stress in experimental liver fibrosis. Hepatol Res 28: 207–215. 15040961 10.1016/j.hepres.2003.11.012

[43] Das J, Roy A, Sil PC (2012) Mechanism of the protective action of taurine in toxin and drug induced organ pathophysiology and diabetic complications: a review. Food Funct 3: 1251–1264. 22930035 10.1039/c2fo30117b

[44] Murphy GM, Signer E (1974) Bile acid metabolism in infants and children. Gut 15: 151–163. 4595082 10.1136/gut.15.2.151 PMC1412900

[45] Hardison WG, Proffitt JH (1977) Influence of hepatic taurine concentration on bile acid conjugation with taurine. Am J Physiol 232: E75–79. 835705 10.1152/ajpendo.1977.232.1.E75

[46] Hardison WG (1978) Hepatic taurine concentration and dietary taurine as regulators of bile acid conjugation with taurine. Gastroenterology 75: 71–75. 401099

[47] Hofmann AF (1999) The continuing importance of bile acids in liver and intestinal disease. Arch Intern Med 159: 2647–2658. 10597755 10.1001/archinte.159.22.2647

[48] Staels B, Fonseca VA (2009) Bile acids and metabolic regulation: mechanisms and clinical responses to bile acid sequestration. Diabetes Care 32: S237–245. 19875558 10.2337/dc09-S355 PMC2811459

