# Supplementary material for: Mass Spectrometry-Based Metabolite Profiling in the Mouse Liver following Exposure to Ultraviolet B Radiation
Source: PLoS One. 2014 Oct 2;9(10):e109479. doi: 10.1371/journal.pone.0109479 (PMC4183543; doi:10.1371/journal.pone.0109479)
Supplement: File S1 — Supporting information. Figure S1. OPLS-DA score plot derived from GC-TOF-MS (A, B), UPLC-Q-TOF-MS (C, D) and nanomate LTQ-MS (E) data sets for MW (A, C), DM (B, D) and lipid (E) extracts of mouse liver tissue after the exposure to UVB radiation for 6 weeks. ▪ - normal, • – UVB. Table S1. Summary of parameters for assessment of the quality of PLS-DA models (DOC) [file pone.0109479.s001.doc]

**Supporting Information (SI)**


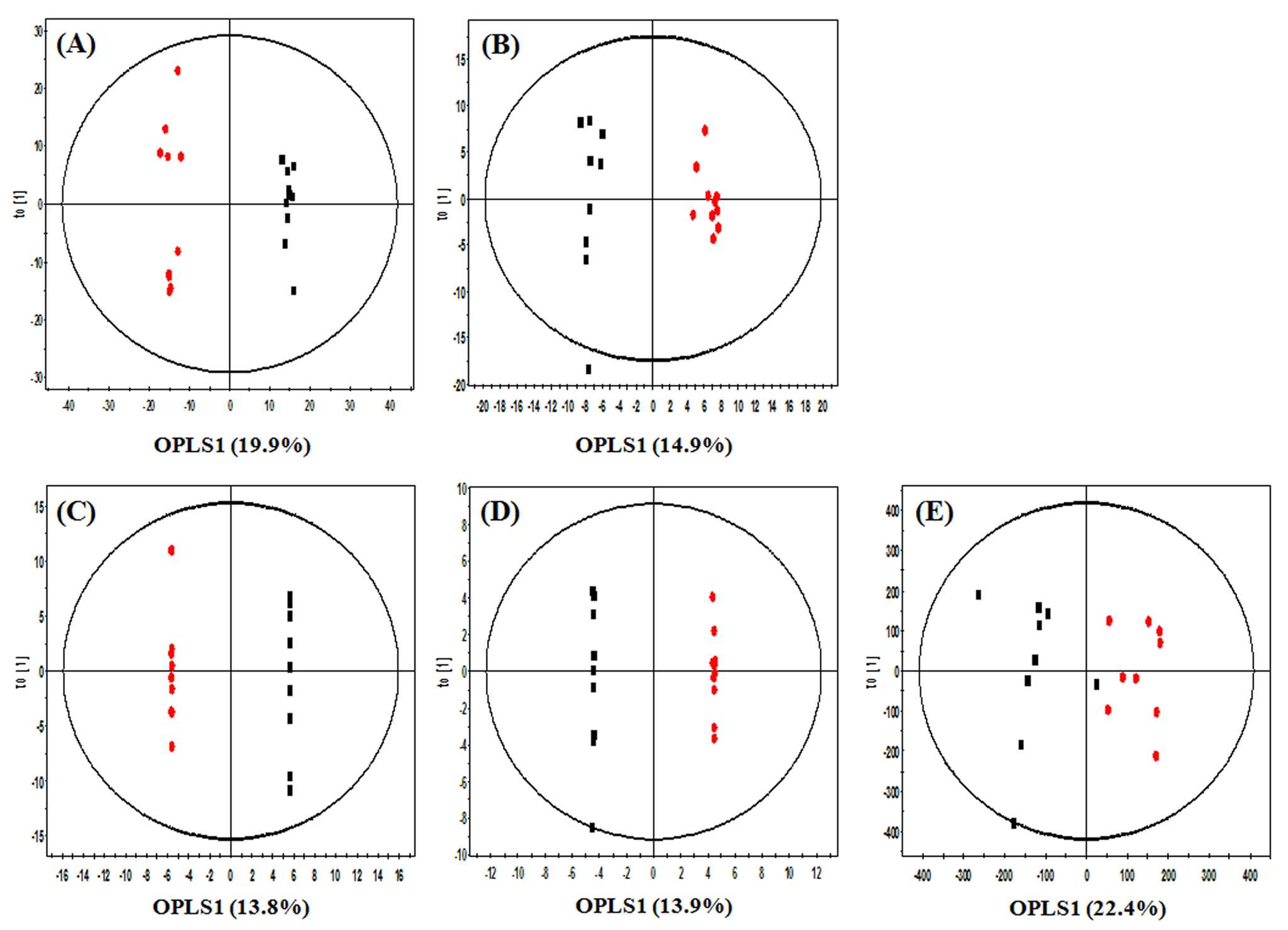


**Figure S1 in File S1**. OPLS-DA score plot derived from GC-TOF-MS (A, B), UPLC-Q-TOF-MS (C, D) and nanomate LTQ-MS (E) data sets for MW (A, C), DM (B, D) and lipid (E) extracts of mouse liver tissue after the exposure to UVB radiation for 6 weeks. ■ - normal, ● - UVB

**Table S1 in File S1. Summary of parameters for assessment of the quality of PLS-DA models**

|  | **Extracts** | **R^2^X_cum_*^a^*** | **R^2^Y_cum_*^a^*** | **Q^2^Y_cum_*^b^*** | ***P^c^*** |
| --- | --- | --- | --- | --- | --- |
| **GC-TOF-MS** | **MW^d^** | 0.357 | 0.978 | 0.827 | < 0.001 |
|  | **DM^e^** | 0.656 | 0.989 | 0.856 | 0.153 |
| **UPLC-Q-TOF-MS** | **MW** | 0.421 | 0.995 | 0.811 | 0.053 |
|  | **DM** | 0.380 | 0.991 | 0.821 | 0.031 |
| **Nanomate LTQ-MS** | **Lipid^f^** | 0.309 | 0.904 | 0.549 | 0.109 |

*^a^*R^2^X_cum_ and R^2^Y_cum_ are the cumulative modeled variations in the X and Y matrices, respectively.

*^b^*Q^2^Y_cum_ is the cumulative predicted variation in the Y matrix.

*^c^P* is the *p* value obtained from cross validation ANOVA of PLS-DA.

^d^MW, methanol/water (1:1, v/v)

^e^DM, dichloromethane/methanol (3:1, v/v)

^f^The lipid extract for nanomate LTQ-MS analysis was prepared as mentioned in M&M section
